# Assessment of CPS + EG, Neo-Bioscore and Modified Neo-Bioscore in Breast Cancer Patients Treated With Preoperative Systemic Therapy: A Multicenter Cohort Study

**DOI:** 10.3389/fonc.2021.606477

**Published:** 2021-03-16

**Authors:** Ling Xu, Yinhua Liu, Zhimin Fan, Zefei Jiang, Yunjiang Liu, Rui Ling, Jianguo Zhang, Zhigang Yu, Feng Jin, Chuan Wang, Shude Cui, Shu Wang, Dahua Mao, Bing Han, Tao Wang, Geng Zhang, Ting Wang, Baoliang Guo, Lixiang Yu, Yingying Xu, Fangmeng Fu, Zhenzhen Liu, Siyuan Wang, Ke Luo, Qian Xiang, Zhuo Zhang, Qianxin Liu, Bin Zhou, Zhaorui Liu, Chao Ma, Weiwei Tong, Jie Mao, Xuening Duan, Yimin Cui

**Affiliations:** ^1^ Breast Disease Center, Peking University First Hospital, Beijing, China; ^2^ Department of Breast Surgery, First Hospital of Jilin University, Changchun, China; ^3^ Department of Breast Cancer, Affiliated Hospital of Academy of Military Medical Sciences, Beijing, China; ^4^ Hebei Breast Cancer Center, The 4th Hospital of Hebei Medical University, Shijiazhuang, China; ^5^ Department of Thyroid, Breast and Vascular Surgery, Xijing Hospital, Air Force (Military) Medical University, Xi’an, China; ^6^ Breast Disease Department, Second Affiliated Hospital of Harbin Medical University, Harbin, China; ^7^ Department of Breast Surgery, Second Hospital of Shandong University, Jinan, China; ^8^ Department of Breast Surgery, The First Hospital of China Medical University, Shenyang, China; ^9^ Department of Breast Surgery, Affiliated Union Hospital of Fujian Medical University, Fuzhou, China; ^10^ Department of Breast Surgery, Affiliated Tumor Hospital of Zhengzhou University, Zhengzhou, China; ^11^ Breast Center, Peking University People’s Hospital, Beijing, China; ^12^ Department of Breast Surgery, The Affiliated Hospital of Guizhou Medical University, Guiyang, China; ^13^ Department of Pharmacy, Peking University First Hospital, Beijing, China; ^14^ Institute of Mental Health, Peking University, Beijing, China; ^15^ Gennlife (Beijing) Technology Co., Ltd, Beijing, China

**Keywords:** preoperative systemic therapy, CPS + EG, Neo-Bioscore, HER2, breast cancer prognosis

## Abstract

This study was to assess the prognosis stratification of the clinical-pathologic staging system incorporating estrogen receptor (ER)-negative disease, the nuclear grade 3 tumor pathology (CPS + EG), Neo-Bioscore, and a modified Neo-Bioscore system in breast cancer patients after preoperative systemic therapy (PST). A retrospective multicenter cohort study was conducted from 12 participating hospitals’ databases from 2006 to 2015. Five-year disease free survival (DFS), disease specific survival (DSS), and overall survival (OS) were calculated using Kaplan–Meier Method. Area under the curve (AUC) of the three staging systems was compared. Wald test and maximum likelihood estimates in Cox proportional hazards model were used for multivariate analysis. A total of 1,077 patients were enrolled. The CPS + EG, Neo-Bioscore, and modified Neo-Bioscore could all stratify the DFS, DSS, and OS (all P < 0.001). While in the same stratum of Neo-Bioscore scores 2 and 3, the HER2-positive patients without trastuzumab therapy had much poorer DSS (P = 0.013 and P values < 0.01, respectively) as compared to HER2-positive patients with trastuzumab therapy and HER2-negative patients. Only the modified Neo-Bioscore had a significantly higher stratification of 5-year DSS than PS (AUC 0.79 *vs*. 0.65, P = 0.03). So, the modified Neo-Bioscore could circumvent the limitation of CPS + EG or Neo-Bioscore.

**Clinical Trial Registration:**

ClinicalTrials.gov, identifier NCT03437837.

## Introduction

Assessment of breast cancer prognosis is critical for clinicians to make therapeutic decision. Previous studies demonstrated that the same chemotherapy regimen used as preoperative systemic therapy (PST) or adjuvant therapy in individual studies did not significantly improve prognosis of the patients’ survival (1, 2). However, pooled analysis showed that patients who achieved a pathological complete response (pCR), defined as the histologically absence of invasive cancer cells in the breast and axillary nodes (ypT0/is ypN0), significantly had superior disease free survival (DFS) after PST, as compared with patients who had residual disease (3). These studies indicated that patient’s responses to PST can predict the disease outcomes, and the residual cancer burden (RCB) can be used to stratify the prognosis of patients after PST (4). On the other hand, most of the patients with hormone receptor (HR)-positive, human epidermal growth factor receptor 2 (HER2)-negative subtypes still had a favorable prognosis although they achieved a low pCR rate (5–7). As demonstrated from recent meta-analysis, pCR was a less strong prognostic factor in luminal type breast cancers, and the prognosis of non-pCR patients varied a lot (3, 8). These studies suggest that the prediction, based primarily on final pathologic assessment of post-treatment cancer residues in the breast and axilla, is not reliable, and a better prognostic approach that integrates biologic markers into clinical and pathological staging systems is needed to improve the prediction of non-pCR patients and some of pCR patients as well for development of therapeutic strategy for postoperative treatments.

The clinical-pathologic staging system incorporating estrogen receptor (ER)-negative disease, the nuclear grade 3 tumor pathology (CPS + EG staging system) (9, 10) and the updated Neo-Bioscore (11) are two score systems that incorporate aspects of tumor biology into staging system. The Neo-Bioscore was developed to validate the CPS + EG staging system using a new definition of ER positivity, and it also incorporated the HER2 status into the previously developed CPS + EG staging system. It can better stratify disease specific survival (DSS) for patients who didn’t achieve pCR. Recent studies have demonstrated that treatment of HER2-positive tumors with trastuzumab has been shown to improve survival in the adjuvant (12, 13) and neoadjuvant setting (14–16). Thus, HER2-negativity has been assigned as an unfavorable prognostic factor on the basis that patients with HER2-positive tumors are routinely treated with trastuzumab and have better prognoses in the Neo-Bioscore prognostic stratification system. This is the same criterion as the prognostic stage in the eighth American Joint Committee on Cancer (AJCC) (17). Interestingly, the distribution of breast cancer subtypes varies within the broad racial/ethnic groups. It appears that Korean, Filipina, Chinese, and Southeast Asian women had a higher incidence of HR-negative/HER2-positive breast cancers compared with non-Hispanic white women (18). Unlike the routine administration of trastuzumab for HER2-positive patients in the United States and other developed countries, most of HER2-positive breast cancer patients could not access trastuzumab because of expensive medical cost and patient’s financial hardship in China. In addition, some of HER2-positive patients who were initially treated with trastuzumab had to withdraw the therapy because of cardiac, no-cardiac toxicity, unfavorable compliance or lack of response during PST. Because both CPS + EG and Neo-Bioscore systems have not incorporated the HER2-positive patients without trastuzumab treatment, the potential to predict the outcome of HER2-positive patients treated without trastuzumab is largely limited. Thus, an accurate prediction for those subgroups of HER2-positive patients needs to be developed.

Here, we conducted a retrospective multicenter cohort study in patients with primary invasive breast cancers who underwent PST and surgery. Our objective of this study was to validate CPS + EG and Neo-Bioscore system and determine the accuracy of prediction of not only DSS, but also DFS and overall survival (OS). We also developed a modified Neo-Bioscore system to stratify the prognosis after PST in more detail.

## Material and Methods

### Patients and Study Design

The study was approved by the central ethics committee at the Peking University First Hospital. All breast cancer patients who met the inclusion criteria were recruited from participating hospitals’ databases from 2006 to 2015. The detailed protocol was published elsewhere (19). The data was recorded by professional clinicians and double-checked by independent research staff for accuracy. The retrospective data was retrieved from the hospitalization and follow-up patient databases from multiple institutes or hospitals.

### Treatment and Standard Procedures

All patients received the first-line of taxanes (T)- and/or anthraclines (A)-based neoadjuvant regimen and other interventions as standard procedures, as described previously (19).

### Statistical Evaluation

The CPS + EG score and Neo-Bioscore were determined for each patient as previously reported (9, 11). Considering HER2-positive patients without trastuzumab therapy as a poor risk factor, we assigned two scores in our modified Neo-Bioscore staging system. The detailed staging systems were summarized in **Table 1**. The Kaplan–Meier method was used to calculate five-year DFS, DSS, and OS for patients’ sub-grouped with multiple staging systems: (1) pretreatment clinical stage (CS), (2) post-treatment pathological stage (PS), (3) CPS + EG score, (4) Neo-Bioscore, and (5) modified Neo-Bioscore. Within each staging system, DFS, DSS, and OS among subgroups were compared using the log-rank test. Area under the curve (AUC) was calculated for the multiple staging systems and compared using the time-dependent ROC package (20). Wald test and maximum likelihood estimates (MLE) in Cox proportional hazards model for DFS, DSS, and OS were used to estimate hazard ratios when the covariates of age, menopause, progesterone receptor (PR), and Ki67 were included together with prognostic scores of the CPS + EG, Neo-Bioscore, or modified Neo-Bioscore staging system, respectively.

**Table 1:** Point Assignment for the CPS + EG, Neo-Bioscore, and Modified Neo-Bioscore Staging Systems.

Cancer Stage	CPS + EG Score	Neo-Bioscore (7 points)	Modified Neo-Bioscore (8 points)
Pretreatment Clinical Stage (CS)			
I	0	0	0
IIA	0	0	0
IIB	1	1	1
IIIA	1	1	1
IIIB	2	2	2
IIIC	2	2	2
Post-treatment Pathologic Stage (PS)			
0	0	0	0
I	0	0	0
IIA	1	1	1
IIB	1	1	1
IIIA	1	1	1
IIIB	1	1	1
IIIC	2	2	2
Tumor Marker			
ER negative	1	1	1
Grade 3	1	1	1
HER2 negative		1	1
HER2 positive & no trastuzumab			2

CPS + EG, clinical-pathologic staging system incorporating estrogen receptor-negative disease and nuclear grade 3 tumor pathology; ER, estrogen receptor; PR, progesterone receptor; HER2, human epidermal growth factor receptor 2.

## Results

### Patient and Tumor Characteristics

**Figure 1:**
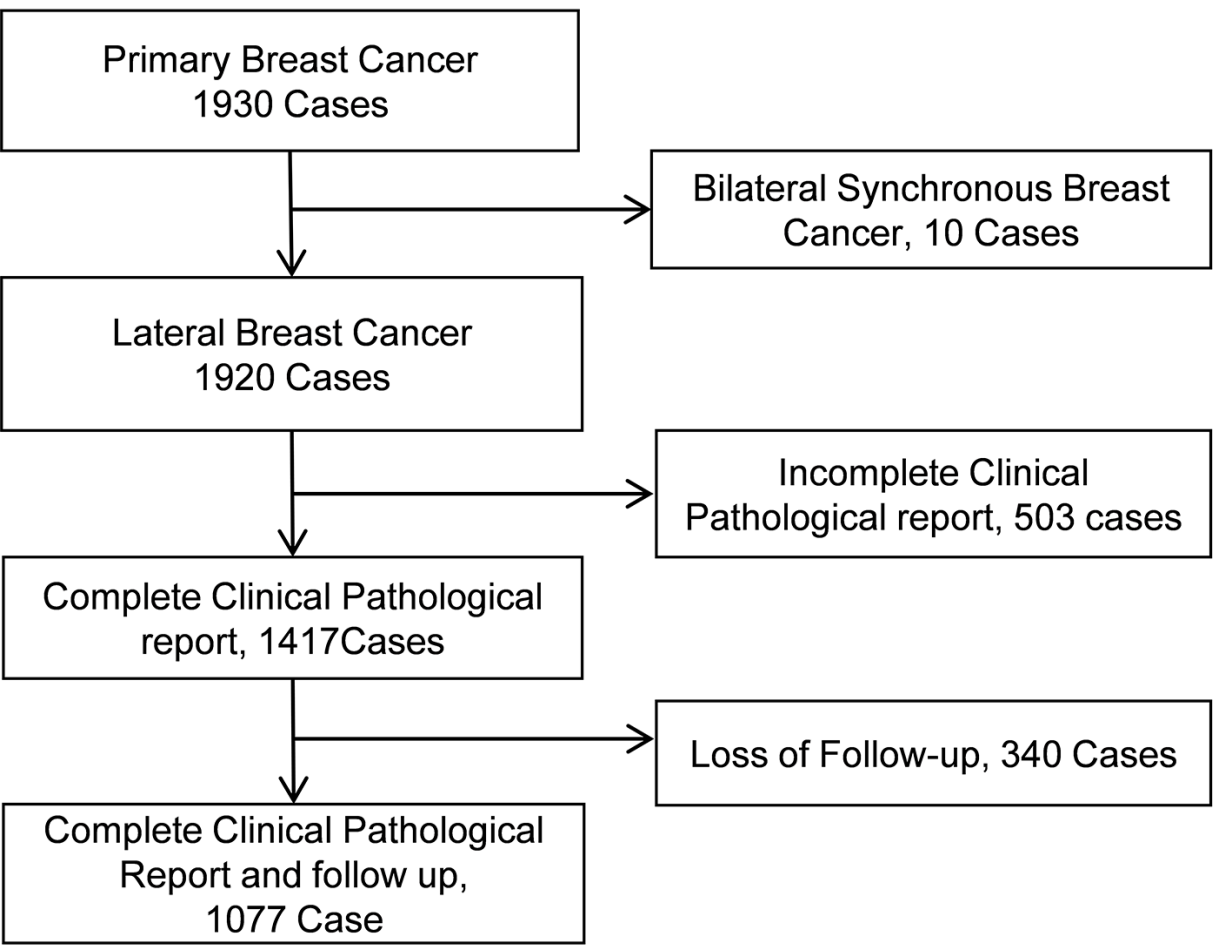
Flow of participants.

A total 1,930 patients with primary breast cancer from 12 top hospitals in China were recruited, of which 1,077 cases met the inclusion criteria and therefore enrolled in this study. A total of 853 patients were excluded from the study (**Figure 1**). Of the 1,077 enrolled patients, pre-menopausal status was in 589 cases (54.7%) and post-menopausal status was in 488 patients (45.3%). The median age at the time of diagnosis was 49 (range 22–74) years old (**Table 2**). The ER status was lower than 1% in 445 patients (41.3%) and 1% or higher in 632 cases (58.7%). A total of 315 patients (29.2%) were HER2 positive; 45.1% of whom received trastuzumab as a component of their PST regimen and consecutive to 1 year, and 54.9% of HER2-positive case did not receive trastuzumab treatment. A total of 762 cases (70.8%) were HER2 negative. A total of 70 cases (6.5%) had clinical stage I disease, 717 patients (66.5%) had clinical stage II disease (29.5% IIA, 37.0% IIB), and 290 cases (26.9%) had clinical stage III disease (16.2% IIIA, 5.4% IIIB, 5.4% IIIC). A total of 166 patients (15.4%) achieved pCR, and 911 cases (84.6%) were non-pCR after PST. Of 911 patients with residual tumor, pathologic stage I was in 264 cases (29.0%), IIA was in 261 cases (28.6%), IIB was in 128 patients (14.1%), IIIA was in 156 patients (17.1%), and IIIB was in 102 cases (11.2%). The median follow-up time was 45 months (range, 11–107 months). The estimated 5-year DFS rate, 5-year DSS rate, and 5-year OS rate for the entire study population were 85.8% (95% CI, 82.9–88.3%), 90.9% (95% CI, 88.4–92.9%), and 89.1% (95% CI, 86.2–91.4%), respectively.

**Table 2:** Cohort Characteristics of Patients (*N* = 1,077) with Primary Breast Cancer.

Variables	*N* = 1077	%	
Age (median, range)	49 (22–74)	
Menopausal status		
Pre-menopause	589	54.7
Post-menopause	488	45.3
Tumor histological grade		
I	46	4.3
II	702	65.2
III	329	30.5
ER status		
Positive	632	58.7
Negative	445	41.3
PR status		
Positive	585	54.3
Negative	492	45.7
HER2 status		
Positive	315	29.2
Negative	762	70.8
Ki 67		
≤14%	148	13.7
>14%	845	78.5
Unknown	84	7.8
Trastuzumab therapy in HER2-positive patients		
Administrated	142	45.1
Without administration	173	54.9
Breast operation		
Lumpectomy	294	27.3
Mastectomy	783	72.7
CS		
I	70	6.5
IIA	318	29.5
IIB	399	37.0
IIIA	174	16.2
IIIB	58	5.4
IIIC	58	5.4
PS		
0	166	15.4
I	264	24.5
IIA	261	24.2
IIB	128	11.9
IIIA	156	14.5
IIIB	10	0.9
IIIC	92	8.5
CPS + EG score		
0	100	9.3
1	212	19.7
2	387	35.9
3	240	22.3
4	111	10.3
5	27	2.5
6	0	0.0
Neo-Bioscore		
0	27	2.5
1	142	13.2
2	236	21.9
3	363	33.7
4	217	20.2
5	76	7.1
6	16	1.5
7	0	0.0
Modified Neo-Bioscore		
0	17	1.6
1	115	10.7
2	194	18.0
3	345	32.0
4	238	22.1
5	113	10.5
6	47	4.4
7	8	0.7
8	0	0.0

ER, estrogen receptor; PR, progesterone receptor; HER2, human epidermal growth factor receptor 2; CS, Pretreatment clinical stage; PS, Post-treatment pathologic stage; CPS+EG, clinical-pathologic staging system incorporating estrogen receptor-negative disease and nuclear grade 3 tumor pathology.

### Five-Year DFS, DSS, and OS Outcomes by CPS + EG and Neo-Bioscore Staging Systems

**Figure 2:**
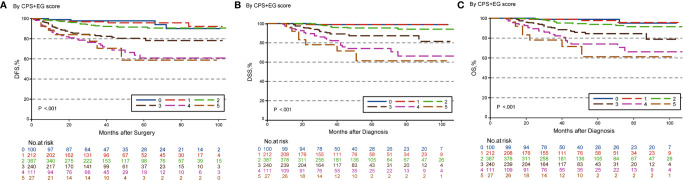
Kaplan–Meier survival curves determined by CPS + EG in patients with breast cancer receiving preoperative systemic therapy (PST). **(A)** Disease free survival (DFS); **(B)** Disease specific survival (DSS); **(C)** Overall survival (OS).

**Figure 3:**
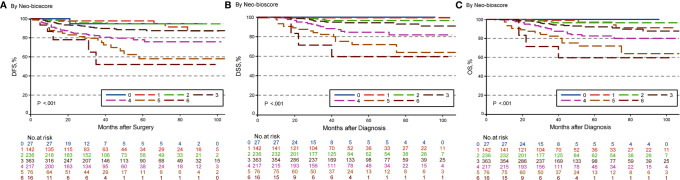
Kaplan–Meier survival curves determined by Neo-Bioscore in patients with breast cancer receiving preoperative systemic therapy (PST). **(A)** Disease free survival (DFS); **(B)** Disease specific survival (DSS); **(C)** Overall survival (OS).

**Figure 4:**
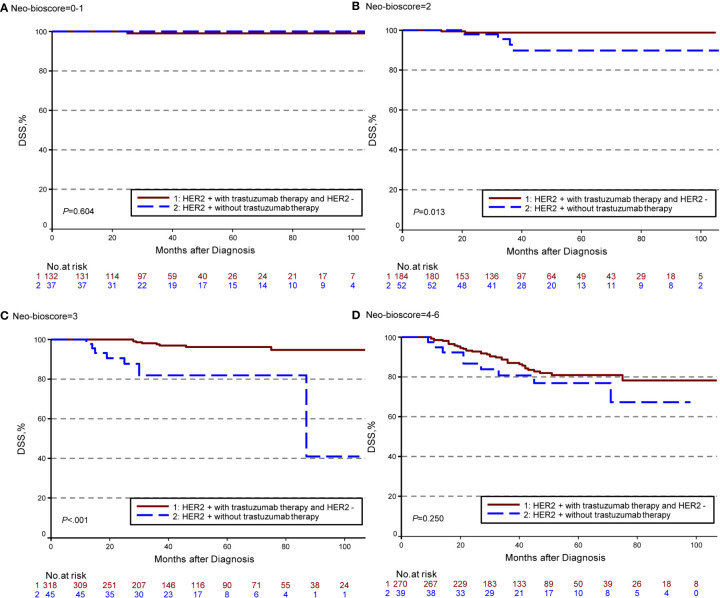
Kaplan–Meier survival curves for disease specific survival (DSS) determined by Neo Bioscore staging system for different HER2 status subgroup. Group 1: HER2-positive patients with trastuzumab therapy and HER2-negative patients; Group 2: HER2-positive patients without trastuzumab therapy. **(A)** DSS of different HER2 status subgroup in Neo-Bioscore scores 0 and 1; **(B)** DSS of different HER2 status subgroup in Neo-Bioscore score 2; **(C)** DSS of different HER2 status subgroup in Neo-Bioscore score 3; **(D)** DSS of different HER2 status subgroup in Neo-Bioscore scores 4, 5, and 6.

**Figure 5:**
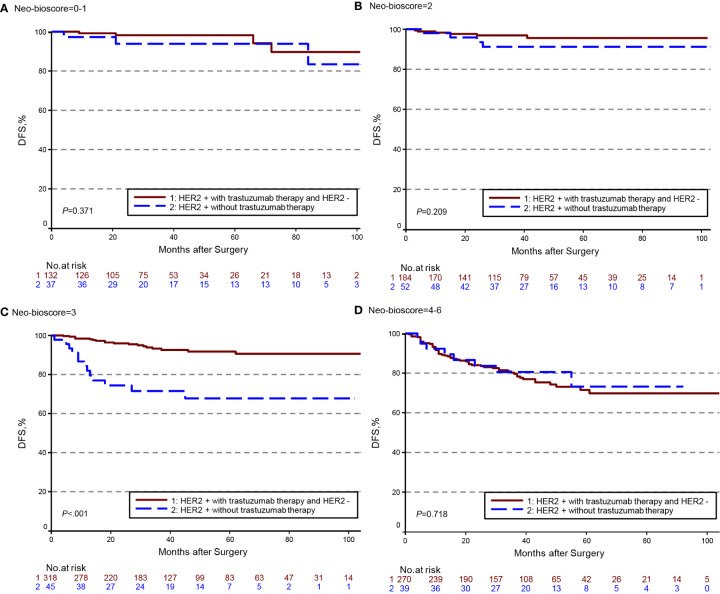
Kaplan–Meier survival curves for disease free survival (DFS) determined by the Neo-Bioscore staging system for different HER2 status subgroup. Group 1: HER2-positive patients with trastuzumab therapy and HER2-negative patients; Group 2: HER2-positive patients without trastuzumab therapy. **(A)** DFS of different HER2 status subgroup in Neo-Bioscore scores 0 and 1; **(B)** DFS of different HER2 status subgroup in Neo-Bioscore score 2; **(C)** DFS of different HER2 status subgroup in Neo-Bioscore score 3; **(D)** DFS of different HER2 status subgroup in Neo-Bioscore scores 4, 5, and 6.

**Figure 6:**
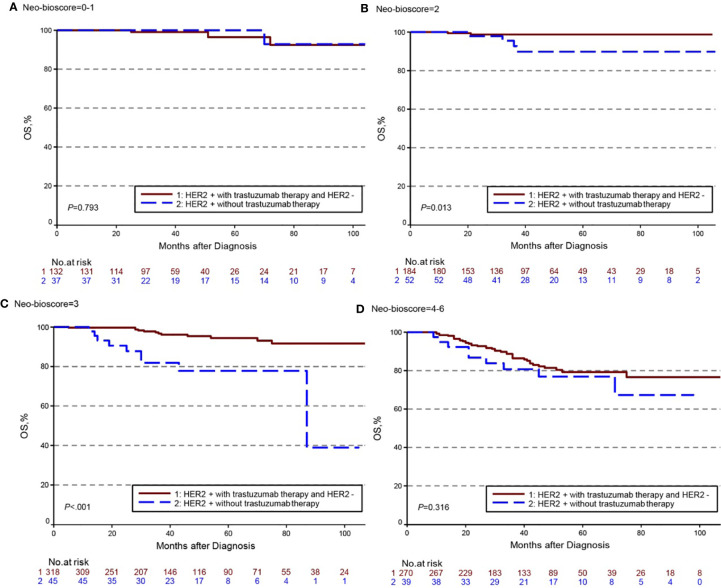
Kaplan–Meier survival curves for overall survival (OS) determined by the Neo-Bioscore staging system for different HER2 status subgroup. Group 1: HER2-positive patients with trastuzumab therapy and HER2-negative patients; Group 2: HER2-positive patients without trastuzumab therapy. **(A)** OS of different HER2 status subgroup in Neo-Bioscore scores 0 and 1; **(B)** OS of different HER2 status subgroup in Neo-Bioscore score 2; **(C)** OS of different HER2 status subgroup in Neo-Bioscore score 3; **(D)** OS of different HER2 status subgroup in Neo-Bioscore scores 4, 5, and 6.

The estimated 5-year DFS, DSS, and OS outcomes by clinical stage, pathologic stage, CPS + EG staing system, and Neo-Bioscore were summarized in **Table 1**. Because a small number of patients had advanced stagings of CPS + EG and Neo-Bioscore, they were combined with either staging 5 or 6 or 7. The CPS + EG score and Neo-Bioscore staging systems for each patient were determined according to the previously published staging system (10, 11). Five-year DSS, DFS, and OS outcomes stratified by the CPS + EG scores or the Neo-Bioscore scores are shown in **Figures 2** and **3**, respectively. Both staging systems of CPS + EG and Neo-Bioscore were significantly associated with DFS, DSS, and OS. Five-year DFS decreased in a step-wise fashion with increasing staging score from 94.03% for score 0 of CPS + EG and 94.74% for score 0 of Neo-Bioscore to 58.77 and 56.69% for advanced stage scores, respectively (**Figure 2A** and **Figure 3A**). Similar step-wise decreases in DSS and OS were seen in the advanced scores for CPS + EG, and Neo-Bioscore staging systems too (**Figures 2B, C**, and **3B, C**). Because nearly 45.9% of HER2-positive patients received trastuzumab-containing therapy, and the other 54.1% of HER2-positive were treated in the absence of trastuzumab, we evaluated the DSS, DFS, and OS outcomes of breast cancer patients stratified by HER2 status in the presence or absence of trastuzumab treatment. Interestingly, 5-year DSS, DFS, and OS for Neo-Bioscore score 3 were all reduced by 32–35% in the HER2-positive patients with the absence of trastuzumab treatment as compared to the same score HER2-positive patients with trastuzumab therapy and HER2-negative patients (**Figures 4C**, **5C**, and **6C**, with all *P* < 0.001). In addition, 5-year DSS and OS for Neo-Bioscore score 2 were also significantly reduced in the HER2-positive patients without trastuzumab treatment (**Figure 4B** and **Figure 6B**, both *P* = 0.013).

**Figure 7:**
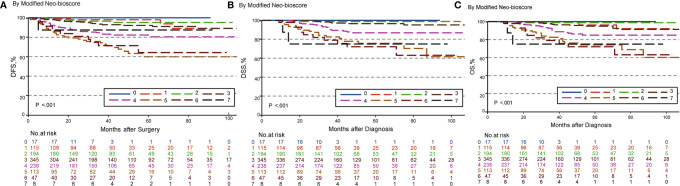
Kaplan–Meier survival curves determined by modified Neo-Bioscore in patients with breast cancer receiving preoperative systemic therapy (PST). **(A)** Disease free survival (DFS); **(B)** Disease specific survival (DSS); **(C)** Overall survival (OS).

**Figure 8:**
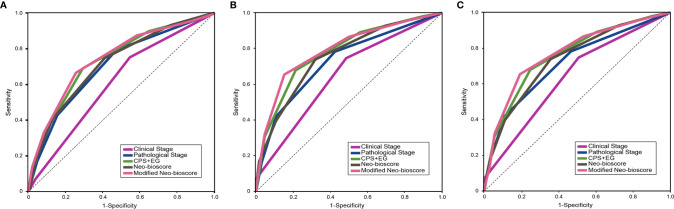
Survival data assessed using the areas under the curves (AUCs) for the pretreatment clinical stage (CS), post-treatment pathological stage (PS), CPS + EG, Neo-Bioscore, and modified Neo-Bioscore. **(A)** Five-year disease free survival (DFS) assessed using AUCs in multiple staging systems; **(B)** Five-year disease specific survival (DSS) assessed using AUCs in multiple staging systems; **(C)** Five-year overall survival (OS) assessed using AUCs in multiple staging systems.

To improve the accuracy of prediction of HER2-positive patients without trastuzumab therapy, we developed a novel staging system, a modified Neo-Bioscore, by adding a point to the Neo-Bioscore for HER2-positive patients without trastuzumab therapy (**Table 1**). We assume that the modified Neo-Bioscore would amend the limitation of CPS + EG and Neo-Bioscore staging systems and provide a better prediction for HER2-positive patients in the absence of standard trastuzumab therapy. The estimated 5-year DFS, DSS, and OS outcomes by the modified Neo-Bioscore were summarized in **Table 3**, and advanced stagings were combined with either staging 6, 7, or 8 because of the small number patients. As shown in **Figure 7**, our modified New-Bioscore score system was also able to predict the outcomes of DFS, DSS, and OS stratified by the modified breast cancer scores (all *P* values <0.001). We assessed the survival data by area under the curve (AUC) in multiple staging system and found that three staging scores of CPS + EG, Neo-Bioscore, and modified Neo-Bioscore had similar prognostic value for 5-year DFS (**Figure 8**). In addition, three staging scores of CPS + EG, Neo-Bioscore, and modified Neo-Bioscore had much better prognosis than CS for DFS, DSS, and OS (all *P* values <0.001) (**Table 4**). The modified Neo-Bioscore had a 5-year DSS AUC that was significantly higher than PS (0.79 *vs* 0.73, *P* value = 0.03), whereas neither CPS + EG (0.78 *vs* 0.73, *P* = 0.1) nor Neo-Bioscore staging system (0.76 *vs* 0.73, *P* = 0.39) had a significant better prediction of 5-year DSS than PS. Neither Neo-Bioscore nor the modified Neo-Bioscore demonstrated an improved discrimination AUC of DFS, DSS, and OS as compared with CPS + EG score. At 5 years, the modified Neo-Bioscore system had a DSS AUC of 0.79 (95% CI 0.74–0.84) that was comparable to the Neo-Bioscore staging system [0.76 (95% CI 0.70–0.81)].

**Table 3:** Five-Year DFS, DSS, and OS Outcomes by Clinical Stage, Pathologic Stage, CPS + EG Staging System, Neo-Bioscore, and Modified Neo-Bioscore.

Staging System	Stages/Scores	DFS (95% CI)	DSS (95% CI)	OS (95% CI)
CS	I (*n* = 70)	92.53(81.26–97.13)	100.00(100–100)	95.83(73.92–99.40)
	IIA (*n* = 318)	93.79(88.98–96.54)	97.96(95.06–99.16)	96.56(92.60–98.42)
	IIB (*n* = 399)	85.08(79.69–89.14)	88.69(82.92–92.59)	87.58(81.86–91.59)
	IIIA (*n* = 174)	84.27(76.47–89.66)	84.81(71.72–92.16)	83.77(73.72–90.22)
	IIIB (*n* = 58)	83.83(69.88–91.69)	86.79(72.17–94.03)	86.79(72.17–94.03)
	IIIC (*n* = 58)	42.53(21.66–62.01)	56.68(30.86–76.02)	56.68(30.86–76.02)
PS	0 (*n* = 166)	94.56(86.56–97.86)	98.26 (93.08–99.57)	95.97 (86.35–98.85)
	I (*n* = 264)	92.48(86.10–95.99)	97.72 (94.58–99.05)	94.84 (87.95–97.84)
	IIA (*n* = 261)	88.72(83.52–92.36)	93.27 (88.32–96.17)	91.82 (86.41–95.14)
	IIB (*n* = 128)	89.86(82.28–94.30)	86.22 (72.96–93.26)	80.81(67.04–89.27)
	IIIA (*n* = 156)	73.03(62.09–81.28)	80.50 (68.49–88.30)	79.98 (68.07–87.83)
CPS+EG	0 (*n* = 100)	94.03 (78.92–98.41)	98.84 (92.03–99.84)	95.04 (78.18–98.95)
	1 (*n* = 212)	92.27(80.19–97.11)	98.94 (95.81–99.74)	95.72 (87.45–98.59)
	2 (*n* = 387)	90.60 (85.85–93.82)	94.06 (89.15–96.79)	92.70 (87.63–95.74)
	3 (*n* = 240)	78.22 (70.18–84.34)	81.44 (65.68–90.46)	78.85 (63.73–88.22)
	4 (*n* = 111)	60.79 (47.53–71.68)	70.69 (57.65–80.38)	70.69 (57.65–80.38)
	5/6 (*n* = 27)^a^	58.77 (27.89–80.15)	61.30 (32.16–80.97)	61.30 (32.16–80.97)
Neo-Bioscore	0 (*n* = 27)	94.74 (68.12–99.24)	100.00 (100.00–100.00)	100.00 (100.00–100.00)
	1 (*n* = 142)	94.62 (82.84–98.39)	99.11 (93.83–99.87)	94.18 (81.31–98.28)
	2 (*n* = 236)	94.65 (90.05–97.15)	96.57 (92.42–98.46)	96.57 (92.42–98.46)
	3 (*n* = 363)	87.47 (82.24–91.24)	92.99 (87.86–96.01)	91.00 (85.61–94.44)
	4 (*n* = 217)	75.82 (67.08–82.54)	81.91 (72.81–88.21)	79.98 (70.62–86.64)
	5/6/7 (*n* = 92)^b^	56.69 (40.56–69.98)	63.21 (44.06–77.36)	63.21 (44.06–77.36)
Modified Neo-Bioscore	0 (*n* = 17)	100.00 (100.00–100.00)	100.00 (100.00–100.00)	100.00 (100.00–100.00)
	1 (*n* = 115)	93.74 (76.61–98.44)	98.91 (92.53–99.85)	92.06 (74.62–97.69)
	2 (*n* = 194)	95.21 (89.80–97.78)	98.83 (95.40–99.71)	98.83 (95.40–99.71)
	3 (*n* = 345)	91.09 (86.02–94.38)	95.20 (89.90–97.75)	92.56 (86.52–95.95)
	4 (*n* = 238)	80.45 (73.12–85.98)	86.83 (80.59–91.17)	84.93 (78.04–89.80)
	5 (*n* = 113)	59.96 (46.60–70.99)	70.36 (54.48–81.59)	68.83 (53.10–80.21)
	6/7/8 (*n* = 55)^c^	67.73 (48.10–81.27)	64.81 (42.44–80.29)	64.81 (42.44–80.29)

CS, pretreatment clinical stage; PS, post-treatment pathological stage; CPS+EG, clinical-pathologic staging system incorporating estrogen receptor-negative disease and nuclear grade 3 tumor pathology; DFS, disease free survival; DSS, disease specific survival; OS overall survival. DFS, DSS, and OS were calculated by K-M method. ^a^scores 5 and 6 of CPS + EG were combined because of small number patients; ^b^scores 5, 6, and 7 of Neo-Bioscore were combined because of small number patients; ^c^scores 6, 7, and 8 of modified Neo-Bioscore were combined because of small number patients.

**Table 4:** Survival Data Assessed by Area Under the Curve (AUC) in Multiple Staging Systems.

	DFS				DSS				OS			
	AUC (95% CI)	*P* ^a^	*P* ^b^	*P* ^c^	AUC (95% CI)	*P* ^a^	*P* ^b^	*P* ^c^	AUC (95% CI)	*P* ^a^	*P* ^b^	*P* ^c^
CS	0.61 (0.56–0.66)				0.65 (0.59–0.71)				0.64 (0.58–0.69)			
PS	0.70 (0.66–0.75)	<0.001			0.73 (0.68–0.78)	0.03			0.72 (0.66–0.77)	0.03		
CPS+EG	0.74 (0.69–0.78)	<0.001	0.16		0.78 (0.73–0.83)	<0.001	0.10		0.76 (0.71–0.81)	<0.001	0.15	
Neo-Bioscore	0.72 (0.67–0.76)	<0.001	0.56	0.07	0.76 (0.70–0.81)	<0.001	0.39	0.06	0.74 (0.68–0.79)	<0.001	0.48	0.08
Modified Neo-Bioscore	0.74 (0.70–0.79)	<0.001	0.09	0.44	0.79 (0.74–0.84)	<0.001	0.03	0.25	0.77 (0.72–0.82)	<0.001	0.07	0.30

CS, pretreatment clinical stage; PS, post-treatment pathological stage; CPS + EG, clinical-pathologic staging system incorporating estrogen receptor-negative disease and nuclear grade 3 tumor pathology; AUC, area under the curve; DFS, disease free survival; DSS, disease specific survival; OS overall survival. ^a^Contrast estimate with CS as reference; ^b^Contrast estimate with PS as reference; ^c^Contrast estimate with CPS + EG as reference.

To determine if the confounding factors of age, menopause, PR, and Ki67 status influence the disease survival of breast cancers, we performed multivariate analyses, using the Wald test and maximum likelihood estimates (MLEs) in Cox proportional hazards model for DFS, DSS, and OS to estimate hazard ratios. As shown in **Table 5**, we found that the menopause status was an independent prognostic factor besides the prognostic scores of the CPS + EG, Neo-Bioscore, or modified Neo-Bioscore staging system scores. The hazard ratios of menopause status for DSS and OS were 2.45 and 2.42, respectively (both *P* < 0.05) in the CPS + EG staging systems, while the hazard ratios of menopause for DSS and OS were 2.67 and 2.61, respectively, in the Neo-Bioscore staging system (both *P* = 0.01), and 2.54, 2.50, respectively, in the modified Neo-Bioscore staging system (both *P* < 0.05). In addition, we noted that age might be an independent factor of prognosis when the modified Neo-Bioscore system was used for prognosis.

**Table 5:** Multivariate Analyses Using Wald test and Maximum Likelihood Estimation (MLE) in Cox Proportional Hazards Model.

Staging System Included	Variables	DFS			DSS			OS		
		Wald test	MLE in the Cox proportional hazards model		Wald test	MLE in the Cox proportional hazards model		Wald test	MLE in the Cox proportional hazards model	
		*P*	HR (95% CI)	*P*	*P*	HR (95% CI)	*P*	*P*	HR (95% CI)	*P*
CPS+EG	Age	0.08	0.98 (0.95–1.00)	0.08	0.12	0.97 (0.4–1.01)	0.12	0.12	0.97 (0.94–1.01)	0.12
	menopause	0.25	1.41 (0.79–2.50)	0.25	0.02	2.45 (1.16–5.15)	0.02	0.01	2.42 (1.19–4.93)	0.01
	PR-Negative	0.69	1.09 (0.72–1.64)	0.69	0.78	1.08 (0.64–1.82)	0.78	0.76	1.08 (0.66–1.78)	0.76
	Ki67 ≥ 14%	0.75	1.11 (0.60–2.04)	0.75	0.73	0.88 (0.41–1.87)	0.73	0.48	0.78 (0.39–1.56)	0.48
	Score	<.0001			<.0001			<.0001		
	1		0.90 (0.26–3.09)	0.87		1.03 (0.09–11.40)	0.98		0.52 (0.07–3.74)	0.52
	2		1.61 (0.55–4.71)	0.38		2.95 (0.38–23.16)	0.30		1.94 (0.44–8.68)	0.38
	3		4.05 (1.41–11.64)	0.01		9.91 (1.31–74.92)	0.03		5.84 (1.35–25.30)	0.02
	4		7.08 (2.44–20.58)	0.00		20.50 (2.71-154.81)	0.00		10.49 (2.41–45.63)	0.00
	5		7.27 (2.06–25.62)	0.00		27.90 (3.33–233.81)	0.00		14.65 (2.93–73.29)	0.00
Neo-Bioscore	Age	0.08	0.97 (0.95–1.00)	0.97	0.11	0.97 (0.93–1.01)	0.11	0.11	0.97 (0.94–1.01)	0.11
	menopause	0.19	1.48 (0.83–2.67)	1.48	0.01	2.67 (1.24–5.77)	0.01	0.01	2.61 (1.26–5.42)	0.01
	PR-Negative	0.29	1.24 (0.83–1.86)	1.24	0.32	1.30 (0.77–2.19)	0.32	0.33	1.27 (0.80–2.08)	0.33
	Ki67 ≥ 14%	0.51	1.23 (0.67–2.27)	1.23	0.91	1.05 (0.49–2.23)	0.91	0.82	0.92 (0.47–1.83)	0.82
	score	<.0001			<.0001			<.0001		
	0		0.11 (0.01–0.98)	0.11		0.00	0.98		0.00	0.98
	1		0.10 (0.03–0.36)	0.10		0.03 (0.00–0.25)	0.00		0.05 (0.01–0.30)	0.00
	2		0.12 (0.04–0.37)	0.12		0.10 (0.03–0.35)	0.00		0.09 (0.03–0.34)	0.00
	3		0.26 (0.10–0.68)	0.26		0.15 (0.05–0.48)	0.00		0.19 (0.06–0.59)	0.00
	4		0.45 (0.17–1.15)	0.44		0.40 (0.13–1.13)	0.08		0.41 (0.14–1.20)	0.10
	5		0.76 (0.28–2.07)	0.76		0.85 (0.28–2.59)	0.77		0.82 (0.27–2.50)	0.73
Modified Neo- Bioscore	Age	0.05	0.97 (0.94–1.00)	0.05	0.07	0.96 (0.93–1.00)	0.07	0.07	0.97 (0.93–1.00)	0.07
	menopause	0.23	1.43 (0.80-2.56)	0.23	0.02	2.54 (1.18–5.45)	0.02	0.01	2.50 (1.21–5.17)	0.01
	PR-Negative	0.76	1.06 (0.71–1.6)	0.76	0.89	1.04 (0.62–1.75)	0.89	0.88	1.04 (0.63–1.70)	0.88
	Ki67 ≥ 14%	0.62	1.17 (0.63–2.16)	0.62	0.74	0.88 (0.41–1.89)	0.74	0.52	0.79 (0.40–1.59)	0.52
	Score	<.0001			<.0001			<.0001		
	0		0.00	0.98		0.00	0.98		0.00	0.98
	1		0.28 (0.03–2.57)	0.26		0.03 (0.00–0.38)	0.01		0.07 (0.01–0.48)	0.00
	2		0.31 (0.04–2.53)	0.27		0.04 (0.00–0.30)	0.00		0.04 (0.01–0.29)	0.00
	3		0.47 (0.06–3.56)	0.46		0.08 (0.02–0.39)	0.00		0.11 (0.02–0.52)	0.00
	4		1.10 (0.15–8.09)	0.93		0.34 (0.08–1.46)	0.15		0.36 (0.08–1.57)	0.18
	5		2.53 (0.34–18.71)	0.36		0.79 (0.18–3.43)	0.76		0.81 (0.19–3.48)	0.77
	6		1.97 (0.25–15.32)	0.52		0.80 (0.17–3.71)	0.77		0.79 (0.17–3.68)	0.76

PR, progesterone receptor; CPS+EG, clinical-pathologic staging system incorporating estrogen receptor–negative disease and nuclear grade 3 tumor pathology; DFS, disease free survival; DSS, disease specific survival; OS, overall survival.

## Discussion

The AJCC published the eighth edition Cancer Staging Manual, and included the traditional anatomic stage groups and the prognostic stage groups which incorporated biomarkers such as ER, PR, HER2 status, and tumor histological grade (17). Both CPS + EG and Neo-Bioscore staging systems are better than the previous CS and PS, which conform to the same point as the AJCC eighth edition that they also take the biomarkers into consideration.

In our previous studies, we demonstrated that both CPS + EG and Neo-Bioscore could predict the disease outcomes well; but they still have obvious limitations (21). The CPS + EG score was developed before the routine test of HER2 status and the use of HER2-targeted therapy. To overcome this limitation, the Neo-Bioscore was developed, which incorporates HER2 status into the CPS + EG. Neo-Bioscore hypothesized that all HER2-positive patients have received anti-HER2 treatment. However, the treatment regimens the patients actually received are not ideal and could have a profound impact on the prognosis (22). For example, HER2 positive could be considered either as a favorable factor if the patients were treated with anti-HER2 treatment, or as a poor prognostic factor if the HER2-positive patients were treated in the absence of trastuzumab therapy (23–25). It is considered in the eighth edition of the AJCC that the prognostic value of these Prognostic Stage Groups relies on populations of patients with breast cancer that have been offered and mostly treated with appropriate endocrine and/or systemic chemotherapy and/or routine use of trastuzumab (17). Data from our hospital breast disease center also showed that the prognostic outcomes (DFS, OS) of the stage I HER2-positive patients was worse than those of the stage II because higher proportion of patients with stage II were treated with trastuzumab than patients with stage I (26).

In fact, insufficient anti-HER2 treatment is common. In this study, we found that of 1,077 patients enrolled from our multicenter approximately 29% (315/1077) of breast patients were HER2-positive; only 45% of them (142/315, HER2+) received trastuzumab, and more than half of HER2-positive cases (173/315, HER2+) were treated in the absence of anti-HER2 treatment. In other countries, patients’ with HER2-positive cancer access to trastuzumab therapy was also limited (27–29). In the United States, for example, a retrospective study that enrolled 915 HER2-positive cases reported that 28% of the HER2-positive patients did not receive anti-HER2 therapy initially (30). Another study reported approximately 41% of 585 American women discontinued trastuzumab therapy mostly because of side effects (31). Early trastuzumab discontinuation was thought to be a powerful independent predictor of cardiac events and clinically significant relapse, and both might contribute to poor survival (31, 32). Thus, the number of HER2-positive patients who do not receive trastuzumab still remains substantial.

We demonstrated that CPS+ EG and Neo-Bioscore staging systems exhibited overall expected outcomes not only of DSS in agreement with other studies (33), but also of DFS and OS which offer more comprehensive information about the long-term prognosis. However, in the same Neo-Bioscore stratum, score of 2 and 3, HER2-positive patients without trastuzumab treatment had obviously worse DSS, DFS, and OS than HER2-negative patients and HER2-positive patients with trastuzumab treatment. However, there were no differences in the higher score group, possibly because of the smaller number of cases or the more important roles of CS and PS staging system played. Our study suggests that an accurate prediction for HER2-positive patients in the absence of trastuzumab treatment needs to be developed for this subgroup of breast cancer patients.

In this study, we developed a modified staging system by assigning HER2-positive without trastuzumab administration as a poorer prognostic factor and gave two points in the modified Neo-Bioscore staging system. We demonstrated that modified Neo-Bioscore had a significant improvement of five-year DSS AUC as compared to PS scoring system (0.79 *vs* 0.73, *P* < 0.05), whereas neither CPS + EG (0.78 *vs* 0.73, *P* = 0.1) nor Neo-Bioscore staging system (0.76 *vs* 0.73, *P* = 0.39) had a significant better prediction of five-year DSS. A possible explanation of failure of the CPS + EG and the Neo-Bioscore prediction is that the characteristics of breast cancer cases in China (*e.g.* age and menopausal status) and the HER2-positive patients without trastuzumab therapy compromised the extent of survival stratification (34). The improvement in the prognosis of breast cancer patients was small; however, we anticipate it may show greater improvement in the predictive value of this novel, modified Neo-Bioscore scoring system once a larger cohort is enrolled in the study.

Unlike the characteristic of breast cancer in the United States, the proportion of young (<40 years old), pre-menopuasal women with breast cancer in China was much higher (34, 35). Pre-menopausal breast cancers were found to comprise a substantially higher proportion of all incident breast cancers in less developed countries (average 47.3%) compared to more developed countries (average 18.5%) (36). Interestingly, multivariate analysis of our study revealed the menopausal status was an independent factor of disease survival prediction besides the scores of the CPS + EG, Neo-Bioscore, modified Neo-Bioscore staging systems. Besides, age also shows a tendency with clinical outcome using modified Neo-Bioscore. Therefore, our future studies will enroll more young breast cancer patients in developing countries to adjust the modified Neo-Bioscore staging system.

The strength of this study was the use of data quality control strategies that are common in the performance of multicenter clinical trials. The study devoted significant attention to data quality at multiple stages, including case ascertainment, data extraction, and data management. However, the study has some limitations which are common in a retrospective design. Higher score groups were ended up with few cases so that the advanced scores were combined for statistical analyses, and a larger cohort study is needed to confirm the finding in future.

We performed a large-scale, multicenter retrospective study and analyzed 1,077 breast cancer patients to validate CPS + EG, Neo-Bioscore, and modified Neo-Bioscore staging systems after PST. We found patients in the absence of trastuzumab therapy had much poorer survival prognosis in the same stratum of Neo-Bioscore scores 2 and 3 as compared to HER2-positive cases with trastuzumab therapy and HER2-negative patients. The modified Neo-Bioscore could circumvent the limitation of CPS + EG or Neo-Bioscore and had a significant improvement of five-year DSS prediction as compared to PS due to mixed trastuzumab therapy in clinical practice. The menopausal status was an independent prognostic factor. Thus, whether menopausal status should be incorporated into the staging system need to be studied and validated with larger samples. Furthermore, it will be our future study to see if other unfavorable prognostic factors, such as insufficient treatment, are incorporated into existing staging system with TNM and biological characteristics, the staging system would have a broader clinical implication in the real world.

## Data Availability Statement

The original contributions presented in the study are included in the article/supplementary material; further inquiries can be directed to the corresponding authors.

## Ethics Statement

The studies involving human participants were reviewed and approved by the Peking University First Hospital Biomedical Research Ethics Committee. Written informed consent for participation was not required for this study in accordance with the national legislation and the institutional requirements.

## Author Contributions

LX, XD, and YC had full access to all the data in the study and take responsibility for the integrity of the data and the accuracy of the data analysis. XD and YC contributed equally to this work. Study concept and design: LX, XD. Acquisition, analysis, or interpretation of data: BZ, BH, TaW, GZ, TiW, BG, LY, YX, FF, ZZL, SYW, KL. Drafting of the manuscript: LX, ZZ, QL. Critical revision of the manuscript for important intellectual content: LX, XD, YC, QX. Statistical analysis: CM, ZRL, LX. Obtained funding: LX, YC. Administrative, technical, or material support: YhL, XD, YC, ZF, ZJ, YJL, RL, JZ, ZY, FJ, CW, SC, SW, DM. Study supervision: YHL, XD, YC. All authors contributed to the article and approved the submitted version.

## Funding

This work was supported by National Key Research and Development Program of China [grant number: 2016YFC0901302, 2016YFC0904900] and Interdisciplinary Clinical Research Project of Peking University First Hospital [grant number: 2019CR38].

## Conflict of Interest

WT and JM were employed by Gennlife (Beijing) Technology Co., Ltd.

The remaining authors declare that the research was conducted in the absence of any commercial or financial relationships that could be construed as a potential conflict of interest.

The reviewer YX declared a shared affiliation, with no collaboration, with several of the authors (LX, YL, SW, SW, QX, ZZ, QL, BZ, ZL, CM, XD, YC) to the handling editor at the time of the review.

## References

[1] Fisher B Bryant J Wolmark N Mamounas E Brown A Fisher ER. Effect of preoperative chemotherapy on the outcome of women with operable breast cancer. J Clin Oncol: Off J Am Soc Clin Oncol (1998) 16(8):2672–85. 10.1200/JCO.1998.16.8.2672 9704717

[2] Early Breast Cancer Trialists’ Collaborative G. Long-term outcomes for neoadjuvant versus adjuvant chemotherapy in early breast cancer: meta-analysis of individual patient data from ten randomised trials. Lancet Oncol (2018) 19(1):27–39. 10.1016/S1470-2045(17)30777-5 29242041 PMC5757427

[3] von Minckwitz G Untch M Blohmer JU Costa SD Eidtmann H Fasching PA. Definition and impact of pathologic complete response on prognosis after neoadjuvant chemotherapy in various intrinsic breast cancer subtypes. J Clin Oncol: Off J Am Soc Clin Oncol (2012) 30(15):1796–804. 10.1200/JCO.2011.38.8595 22508812

[4] Symmans WF Wei C Gould R Yu X Zhang Y Liu M. Long-Term Prognostic Risk After Neoadjuvant Chemotherapy Associated With Residual Cancer Burden and Breast Cancer Subtype. J Clin Oncol: Off J Am Soc Clin Oncol (2017) 35(10):1049–60. 10.1200/JCO.2015.63.1010 PMC5455352 28135148

[5] Mittendorf EA Hunt KK Boughey JC Bassett R Degnim AC Harrell R. Incorporation of Sentinel Lymph Node Metastasis Size Into a Nomogram Predicting Nonsentinel Lymph Node Involvement in Breast Cancer Patients With a Positive Sentinel Lymph Node. Ann Surg (2012) 255(1):109–15. 10.1097/SLA.0b013e318238f461 PMC4760742 22167004

[6] Marmé F Aigner J Bermejo JL Sinn P Sohn C Jäger D. Neoadjuvant epirubicin, gemcitabine and docetaxel for primary breast cancer: Long-term survival data and major prognostic factors based on two consecutive neoadjuvant phase I/II trials. Int J Cancer (2013) 133(4):1006–15. 10.1002/ijc.28094 23400797

[7] Heil J Gondos A Rauch G Marme F Rom J Golatta M. Outcome analysis of patients with primary breast cancer initially treated at a certified academic breast unit. Breast (2012) 21(3):303–8. 10.1016/j.breast.2012.01.009 22310244

[8] Cortazar P Zhang L Untch M Mehta K Costantino J Wolmark N. Meta-analysis Results from the Collaborative Trials in Neoadjuvant Breast Cancer (CTNeoBC). Cancer Res (2012) 72(24 Supplement):S1–11-S1. 10.1158/0008-5472.Sabcs12-S1-11

[9] Jeruss JS Mittendorf EA Tucker SL Gonzalez-Angulo AM Buchholz TA Sahin AA. Combined use of clinical and pathologic staging variables to define outcomes for breast cancer patients treated with neoadjuvant therapy. J Clin Oncol: Off J Am Soc Clin Oncol (2008) 26(2):246–52. 10.1200/JCO.2007.11.5352 18056680

[10] Mittendorf EA Jeruss JS Tucker SL Kolli A Newman LA Gonzalez-Angulo AM. Validation of a novel staging system for disease-specific survival in patients with breast cancer treated with neoadjuvant chemotherapy. J Clin Oncol: Off J Am Soc Clin Oncol (2011) 29(15):1956–62. 10.1200/JCO.2010.31.8469 PMC3107758 21482989

[11] Mittendorf EA Vila J Tucker SL Chavez-MacGregor M Smith BD Symmans WF. The Neo-Bioscore Update for Staging Breast Cancer Treated With Neoadjuvant Chemotherapy: Incorporation of Prognostic Biologic Factors Into Staging After Treatment. JAMA Oncol (2016) 2(7):929–36. 10.1001/jamaoncol.2015.6478 PMC5757376 26986538

[12] Piccart-Gebhart MJ Procter M Leyland-Jones B Goldhirsch A Untch M Smith I. Trastuzumab after adjuvant chemotherapy in HER2-positive breast cancer. N Engl J Med (2005) 353(16):1659–72. 10.1056/NEJMoa052306 16236737

[13] Romond EH Perez EA Bryant J Suman VJ Geyer CE Jr Davidson NE. Trastuzumab plus adjuvant chemotherapy for operable HER2-positive breast cancer. N Engl J Med (2005) 353(16):1673–84. 10.1056/NEJMoa052122 16236738

[14] Mittendorf EA Wu Y Scaltriti M Meric-Bernstam F Hunt KK Dawood S. Loss of HER2 amplification following trastuzumab-based neoadjuvant systemic therapy and survival outcomes. Clin Cancer Res (2009) 15(23):7381–8. 10.1158/1078-0432.CCR-09-1735 PMC2788123 19920100

[15] Buzdar AU Valero V Ibrahim NK Francis D Broglio KR Theriault RL. Neoadjuvant therapy with paclitaxel followed by 5-fluorouracil, epirubicin, and cyclophosphamide chemotherapy and concurrent trastuzumab in human epidermal growth factor receptor 2-positive operable breast cancer: an update of the initial randomized study population and data of additional patients treated with the same regimen. Clin Cancer Res (2007) 13(1):228–33. 10.1158/1078-0432.CCR-06-1345 17200359

[16] Buzdar AU Ibrahim NK Francis D Booser DJ Thomas ES Theriault RL. Significantly higher pathologic complete remission rate after neoadjuvant therapy with trastuzumab, paclitaxel, and epirubicin chemotherapy: results of a randomized trial in human epidermal growth factor receptor 2-positive operable breast cancer. J Clin Oncol: Off J Am Soc Clin Oncol (2005) 23(16):3676–85. 10.1200/JCO.2005.07.032 15738535

[17] Hortobagyi GN Connolly JL D’Orsi CJ. Breast. 8th. Amin MB Edge SB Greene FL, editors. New York: Springer (2017) p. 589–628.

[18] Telli ML Chang ET Kurian AW Keegan TH McClure LA Lichtensztajn D. Asian ethnicity and breast cancer subtypes: a study from the California Cancer Registry. Breast Cancer Res Treat (2011) 127(2):471–8. 10.1007/s10549-010-1173-8 PMC4349378 20957431

[19] Xu L Zhang Z Liu Q Zhou B Liu Y Xiang Q. Validation of CPS+EG, Neo-Bioscore, and modified Neo-Bioscore staging systems after preoperative systemic therapy of breast cancer: Protocol of a retrospective multicenter cohort study in China. Thorac Cancer (2018) 9(11):1565–72. 10.1111/1759-7714.12852 PMC6209787 30296013

[20] Blanche P Dartigues JF Jacqmin-Gadda H. Estimating and comparing time-dependent areas under receiver operating characteristic curves for censored event times with competing risks. Stat Med (2013) 32(30):5381–97. 10.1002/sim.5958 24027076

[21] Xu L Duan X Zhou B Liu Y Ye J Liu Z. Validation of the CPS+EG and Neo-Bioscore staging systems after preoperative systemic therapy for breast cancer in a single center in China. Breast (2018) 40:29–37. 10.1016/j.breast.2018.03.010 29677568

[22] Cossetti RJ Tyldesley SK Speers CH Zheng Y Gelmon KA. Comparison of breast cancer recurrence and outcome patterns between patients treated from 1986 to 1992 and from 2004 to 2008. J Clin Oncol: Off J Am Soc Clin Oncol (2015) 33(1):65–73. 10.1200/JCO.2014.57.2461 25422485

[23] Slamon DJ Godolphin W Jones LA Holt JA Wong SG Keith DE. Studies of the HER-2/neu proto-oncogene in human breast and ovarian cancer. Science (1989) 244(4905):707–12. 10.1126/science.2470152 2470152

[24] Seshadri R Firgaira FA Horsfall DJ McCaul K Setlur V Kitchen P. Clinical significance of HER-2/neu oncogene amplification in primary breast cancer. The South Australian Breast Cancer Study Group. J Clin Oncol: Off J Am Soc Clin Oncol (1993) 11(10):1936–42. 10.1200/JCO.1993.11.10.1936 8105035

[25] Ross JS Fletcher JA. The HER-2/neu oncogene in breast cancer: prognostic factor, predictive factor, and target for therapy. Stem Cells (1998) 16(6):413–28. 10.1002/stem.160413 9831867

[26] Zhou B Xu L Ye J Xin L Duan X Liu Y. The Prognostic Value of the 8th Edition of the American Joint Committee on Cancer (AJCC) Staging System in HER2-Enriched Subtype Breast Cancer, a Retrospective Analysis. Anticancer Res (2017) 37(8):4615–21. 10.21873/anticanres.11862 28739761

[27] Boons CC Wagner C Hugtenburg JG. Guideline Adherence Regarding the Use of Expensive Drugs in Daily Practice: The Examples of Trastuzumab in Breast Cancer and Bortezomib in Multiple Myeloma. Oncol Res Treat (2016) 39(7-8):417–22. 10.1159/000447280 27486994

[28] Neugut AI Hillyer GC Kushi LH Lamerato L Leoce N Ambrosone CB. Non-initiation and early discontinuation of adjuvant trastuzumab in women with localized HER2-positive breast cancer. Breast Cancer (2014) 21(6):780–5. 10.1007/s12282-014-0543-1 PMC4213210 24902664

[29] Coulson SG Kumar VS Manifold IM Hatton MQ Ramakrishnan S Dunn KS. Review of testing and use of adjuvant trastuzumab across a cancer network–are we treating the right patients? Clin Oncol (2010) 22(4):289–93. 10.1016/j.clon.2010.02.011 20347281

[30] DaCosta Byfield S Buck PO Blauer-Peterson C Poston SA DaCosta Byfield S Buck PO. ReCAP: Treatment Patterns and Cost of Care Associated With Initial Therapy Among Patients Diagnosed With Operable Early-Stage Human Epidermal Growth Factor Receptor 2-Overexpressed Breast Cancer in the United States: A Real-World Retrospective Study. J Oncol Pract (2016) 12(2):159–67. 10.1200/JOP.2015.004747 26395563

[31] Wang SY Long JB Hurria A Owusu C Steingart RM Gross CP. Cardiovascular events, early discontinuation of trastuzumab, and their impact on survival. Breast Cancer Res Treat (2014) 146(2):411–9. 10.1007/s10549-014-3029-0 24951268

[32] Gong IY Verma S Yan AT Ko DT Earle CC Tomlinson GA. Long-term cardiovascular outcomes and overall survival of early-stage breast cancer patients with early discontinuation of trastuzumab: a population-based study. Breast Cancer Res Treat (2016) 157(3):535–44. 10.1007/s10549-016-3823-y 27271767

[33] Abdelsattar JM Al-Hilli Z Hoskin TL Heins CN Boughey JC. Validation of the CPS + EG Staging System for Disease-Specific Survival in Breast Cancer Patients Treated with Neoadjuvant Chemotherapy. Ann Surg Oncol (2016) 23(10):3206–11. 10.1245/s10434-016-5324-y 27328945

[34] Li J Zhang BN Fan JH Pang Y Zhang P Wang SL. A nation-wide multicenter 10-year (1999-2008) retrospective clinical epidemiological study of female breast cancer in China. BMC Cancer (2011) 11:364. 10.1186/1471-2407-11-364 21859480 PMC3178543

[35] DeSantis CE Fedewa SA Goding Sauer A Kramer JL Smith RA Jemal A. Breast cancer statistics, 2015: Convergence of incidence rates between black and white women. CA: Cancer J Clin (2016) 66(1):31–42. 10.3322/caac.21320 26513636

[36] Ghiasvand R Adami HO Harirchi I Akrami R Zendehdel K. Higher incidence of premenopausal breast cancer in less developed countries; myth or truth? BMC Cancer (2014) 14(1):343. 10.1186/1471-2407-14-343 24884841 PMC4032450

